# Supplementary material for: The molecular basis of Human FN3K mediated phosphorylation of glycated substrates
Source: Nat Commun. 2025 Jan 22;16:941. doi: 10.1038/s41467-025-56207-z (PMC11754801; doi:10.1038/s41467-025-56207-z)
Supplement: Supplementary file 1 — Reporting Summary [file 41467_2025_56207_MOESM1_ESM.pdf]

nature portfolio

Corresponding author(s): Leemor Joshua-Tor

Last updated by author(s): Dec 3, 2024

Reporting Summary

Nature Portfolio wishes to improve the reproducibility of the work that we publish. This form provides structure for consistency and transparency in reporting. For further information on Nature Portfolio policies, see our Editorial Policies and the Editorial Policy Checklist.

Statistics

For all statistical analyses, confirm that the following items are present in the figure legend, table legend, main text, or Methods section.

n/a	Confirmed	
[ ]	[x]	The exact sample size (*n*) for each experimental group/condition, given as a discrete number and unit of measurement
[ ]	[x]	A statement on whether measurements were taken from distinct samples or whether the same sample was measured repeatedly
[x]	[ ]	The statistical test(s) used AND whether they are one- or two-sided<br>*Only common tests should be described solely by name; describe more complex techniques in the Methods section.*
[x]	[ ]	A description of all covariates tested
[x]	[ ]	A description of any assumptions or corrections, such as tests of normality and adjustment for multiple comparisons
[ ]	[x]	A full description of the statistical parameters including central tendency (e.g. means) or other basic estimates (e.g. regression coefficient) AND variation (e.g. standard deviation) or associated estimates of uncertainty (e.g. confidence intervals)
[x]	[ ]	For null hypothesis testing, the test statistic (e.g. *F*, *t*, *r*) with confidence intervals, effect sizes, degrees of freedom and *P* value noted<br>*Give P values as exact values whenever suitable.*
[x]	[ ]	For Bayesian analysis, information on the choice of priors and Markov chain Monte Carlo settings
[x]	[ ]	For hierarchical and complex designs, identification of the appropriate level for tests and full reporting of outcomes
[x]	[ ]	Estimates of effect sizes (e.g. Cohen's *d*, Pearson's *r*), indicating how they were calculated

Our web collection on statistics for biologists contains articles on many of the points above.

Software and code

Policy information about availability of computer code

Data collection	The X-ray diffraction data for apo, AMPPNP-DMF, ATP-DMF, ADP-DMF (II), and D217S-ATP crystals were collected at the Advanced Photon Source NE-CAT section beamline 24-ID-E at Argonne National Laboratory. X-ray diffraction data for the ADP-DMF(I) structure were collected at beamline 17-ID-1 at NSLS-II at Brookhaven National Laboratory.
Data analysis	Coot(0.9.6), Phenix(1.20.1.4487), PyMol(2.5.5) and GraphPad Prismv(v10) were used for data analysis

For manuscripts utilizing custom algorithms or software that are central to the research but not yet described in published literature, software must be made available to editors and reviewers. We strongly encourage code deposition in a community repository (e.g. GitHub). See the Nature Portfolio guidelines for submitting code & software for further information.

Data

Policy information about availability of data

All manuscripts must include a data availability statement. This statement should provide the following information, where applicable:

- Accession codes, unique identifiers, or web links for publicly available datasets
- A description of any restrictions on data availability
- For clinical datasets or third party data, please ensure that the statement adheres to our policy

The atomic coordinates and structure factors have been deposited in the Protein Data Bank under accession codes 9CX8 for HsFN3K-apo, 9CXV for HsFN3K-ADP-DMF (I), 9CXW for HsFN3K-ADP-DMF (II), 9CXM for the HsFN3K-ATP-DMF, 9CXN for HsFN3K-AMPPNP-DMF and 9CXO for HsFN3K-(D217S)-ATP structure.

Research involving human participants, their data, or biological material

Policy information about studies with human participants or human data. See also policy information about sex, gender (identity/presentation), and sexual orientation and race, ethnicity and racism.

Reporting on sex and gender	n/a
Reporting on race, ethnicity, or other socially relevant groupings	n/a
Population characteristics	n/a
Recruitment	n/a
Ethics oversight	n/a

Note that full information on the approval of the study protocol must also be provided in the manuscript.

Field-specific reporting

Please select the one below that is the best fit for your research. If you are not sure, read the appropriate sections before making your selection.

- [x] Life sciences
- [ ] Behavioural & social sciences
- [ ] Ecological, evolutionary & environmental sciences

For a reference copy of the document with all sections, see nature.com/documents/nr-reporting-summary-flat.pdf

Life sciences study design

All studies must disclose on these points even when the disclosure is negative.

Sample size	sample size was chosen to ensure reproducibility of the results at affordable costs. Atleast 3 replicates were performed for the in-vitro kinase assays.
Data exclusions	no data was excluded from the analysis
Replication	All the details for the replication attempts are mentioned in the methods. All replication were repeated atleast 3 times for kinase assays on DMF, while atleast 2 times for kinase assay involving glycated lysozyme substrate. All replication attempts were successful.
Randomization	n/a
Blinding	Different in-vitro experiments in this study were complex in terms of number of components and organization. So more than one authors designed and analyzed the experiments, while one person performed the experiment. The replicates were performed on different dates to have some experimental/user error in the data.

Reporting for specific materials, systems and methods

We require information from authors about some types of materials, experimental systems and methods used in many studies. Here, indicate whether each material, system or method listed is relevant to your study. If you are not sure if a list item applies to your research, read the appropriate section before selecting a response.

Materials & experimental systems

n/a	Involved in the study	
x		Antibodies
	x	Eukaryotic cell lines
x		Palaeontology and archaeology
x		Animals and other organisms
x		Clinical data
x		Dual use research of concern
x		Plants

Methods

n/a	Involved in the study	
x		ChIP-seq
x		Flow cytometry
x		MRI-based neuroimaging

Eukaryotic cell lines

Policy information about cell lines and Sex and Gender in Research

Cell line source(s)	Sf9 insect cells, LifeTechnologies #11496-015
Authentication	Cell lines were authenticated by the vendors
Mycoplasma contamination	Cell lines were Mycoplasma negative, as tested by the vendor.
Commonly misidentified lines (See ICLAC register)	No commonly misidentified cell lines were included in the study.

Plants

Seed stocks	n/a
Novel plant genotypes	n/a
Authentication	n/a
